# The Matron's Part in the Territorial Army

**Published:** 1908-10-10

**Authors:** 


### Nursing Administration.

### THE MATRON'S PART IN THE TERRITORIAL ARMY.

General interest has been stirred in the nursing organisation of the Territorial Army. The problems relating to it have been encountered with intense energy, and at times so realistic an impression has been produced that it required an effort of the mind to believe that the invading forces were not at the moment encamped on the far side of Oxford Street. The result has been the infusion of an entirely new spirit into a somewhat stale subject. Many influential people held from the beginning that it could serve no good purpose to pledge some 2,700 persons for the future performance of work so nearly akin to their daily duties that no additional training, as in the case of citizen volunteers, was needed before they were called out. The advisory council, charged with the duty of framing regulations for the conduct of the Territorial Nursing Service, has, however, decided otherwise, and a council which has for its secretary Miss Sidney Browne, R.R.C., and numbers among its members Miss McCall Anderson, R.R.C., Miss Cox Davis, Miss Hamilton, Miss Hughes, Miss Ray, and Miss Vernet has strong claims to be listened to with respect. It would seem that while fully recognising the fact that the necessary number of nurse volunteers could be speedily mobilised in case of need by lists supplied from matrons, each pledged to furnish a definite number of suitable people, the council believed that this course would prove a great disappointment to many nurses desirous of taking part in a great national movement, and that it would be inexpedient to waste the opportunity of rousing among nurses at large a due sense of their responsibility to the country. The position of the volunteer matrons under the new scheme is of a totally different character to that of the nurses, inasmuch as it demands from the first distinct services to the force, and is likely to give scope for qualities of the highest order. An able article in the September number of the "Contemporary Review," by Dr. Elizabeth Haldane, LL.D., who is herself a member of the Advisory Council, throws considerable light on the part which the matrons will be called on to play in the development of the Territorial Nursing Service.

"The Territorial Military Hospitals, as has been said, number 23, and for each of these hospitals a matron will be allocated, who will be required to undertake the administration of an establishment of 30 sisters, and 88 nurses. It will be necessary for this prospective matron to study for one week every two years the administrative duties of matrons in large military hospitals. The actual staffing of the 23 hospitals, which will among them have about 12,000 beds, will be one matron, 22 sisters, and 68 staff nurses; but the numbers and ranks of the nurses on the roll will be two matrons, 30 sisters, and 88 staff nurses in order to allow for casualties. . . . As regards the methods of obtaining recruits, the proposal is to have an organising matron at each of the 23 centres, who will keep a register of nurses in her area, *i.e.* the area in which the general hospital is situated. She, it is hoped, will be co-opted on the county association when questions involving nursing matters are being discussed; one can well imagine that her advice would often be useful in discussing future hospital arrangements, since she will probably be the matron of a large and important civil hospital."

It is evident from the above that the post of organising matron under the Territorial Nursing Service will be a post of a very influential character. It is greatly to be desired, although it may not always be feasible, that the organising matron for the area should also be the volunteer matron on whom, in case of necessity, the duty of administration would fall. In this case the immense advantage of knowing her staff, and of being fully acquainted with local conditions would be possessed by the woman called to face this responsibility. She would have had opportunities of proving her own powers of organisation and would commence her duties with the confidence in self which can only be gained by mixing on terms of equality with other administrators.

The main drawback to the scheme is the likelihood that matrons of "large and important civil hospitals" whether in London or in the provinces will consider that they are already amply provided with work and responsibilities without taking on their shoulders new duties of unknown proportions. Should the nursing service of the Territorial Army fail to secure the adhesion of the matrons in the various areas, it will in our judgment result in serious loss to the cause, which must chiefly look to the matrons to develop the desired sense of patriotic responsibility in the nurses. It will also be a loss of opportunity to the matrons themselves. In all questions of public service it holds good that what is gained is equal to what is given. There are too few occasions for the English matron to reveal her powers save within the seclusion of the hospital walls. In the newer countries the matron is a factor in the life of the community, and her position is recognised universally as one of influence and dignity, not merely in her own particular sphere. To come into contact with those who are occupied in other weighty branches of administrative work, and feel a sense of comradeship with people, heavily burdened, it may be, but with different cares, is perhaps the most exhilarating experience which a matron oppressed with conflicting duties can encounter. She is no longer solitary. She is one of a company of fellow citizens. She finds her accumulated knowledge and experience valuable in quite new directions, and it assumes a freshness astonishing in her own eyes. The apparent impossibility of fulfilling public obligations will, it is earnestly to be hoped, melt away before the loyal determination not to fail the country, and when once the cordial co-operation of the matrons has been obtained the future of the new Volunteer Nursing Service will be assured.

